# Effects of predisposing factors on the success and treatment period in vaginismus

**DOI:** 10.5935/1518-0557.20200018

**Published:** 2020

**Authors:** Ali Doğukan Anğın, İsmet Gün, Önder Sakin, Muzaffer Seyhan Çıkman, Süleyman Eserdağ, Pınar Anğın

**Affiliations:** 1 University of Health Sciences, Dr Lütfi Kırdar Kartal Training and Research Hospital, Department of Obstetrics and Gynecology, İstanbul, Turkey; 2 University of Health Sciences, Sultan Abdülhamid Han Training and Research Hospital, Department of Obstetrics and Gynecology, İstanbul, Turkey; 3 Hera Women's Health Center, Sexual Dysfunction and Gynecology Clinic, İstanbul, Turkey; 4 Ümraniye Training and Research Hospital, Department of Obstetrics and Gynecology, İstanbul, Turkey

**Keywords:** vaginismus, dilator, success, prognosis, predisposing

## Abstract

**Objective:**

There are many predisposing factors associated with vaginismus, but there is lack of data in the literature regarding which and how of these factors influence the success rate of treatment. Our aim is to investigate the effects of factors that are considered as predisposing factors for vaginismus on treatment prognosis and success rate, with cognitive-behavioral therapy and desensitization exercises after sexual therapy.

**Methods:**

Patients with vaginismus were divided into three groups. Group 1: patients who successfully completed vaginal penetration exercises after sexual therapy and experienced vaginal sexual intercourse; Group 2: patients who started penetration exercises but could not reach success; Group 3: patients who discontinued treatment before starting exercises. Demographic and sexual parameters were compared between the groups.

**Results:**

There were statistically significant differences between the groups in terms of history of vaginismus in relatives (4.3%, 23% and 35.7%, *p*=0.047, respectively), the unsuccessful therapy history (69%, 61% and 21.4%, *p*=0.014, respectively), and anal and/or oral sex ratios (47.8%, 7.7% and 57.1%, *p*=0.019, respectively). Mean number of sessions were significantly higher in patients saying, “It is my fault” than among those perceiving it as a common problem (10.6±2.9 ve 7.5±5.7, *p*=0.042, respectively), and in patients with sexual disorder in their male partners than those not having any problem (13.3±3.7 ve 8.2±3.7, *p*=0.013, respectively).

**Conclusion:**

Patients are more resistant to treatment if they have a history of vaginismus among relatives or when one of the couple say, it is his or her fault.

## INTRODUCTION

Vaginismus - recurrent or persistent voluntary contractions of the vagina musculature - is one of the most common female psychosexual dysfunctions (Bhatt *et al*., 2017; Melnik *et al*., 2012). It is associated with significant distress and deterioration in quality of life for women. It may lead to several problems such as psychological, psychosomatic and relationship problems. The prevalence of vaginismus in the general population is 1-6%, and this ratio rises to between 5% and 17% in sexual dysfunction clinics (Konkan *et al*., 2012). These ratios may vary among societies and there are studies reporting much higher rates of vaginismus in Turkey (41-58%) (Şafak Öztürk & Arkar, 2017).

Many different predisposing factors have arisen since James Marion Sims (he was the first who coined the term “vaginismus” in 1862) to the present day, with very different theories about how vaginismus occurs; negative perception about sexuality, growing in a conflicting family, sexual problems with the male partner, sexual and physical abuse, iatrogenic traumas (urethral catheter, enema, genital examination), sexual myths, religious conservatism, relationship conflicts, and psychiatric diseases are among them (Konkan *et al*., 2012; Şafak Öztürk & Arkar, 2017; Pacik & Geletta, 2017; Reissing *et al*., 2014). Similarly, there are many techniques described for vaginismus, such as cognitive behavioral therapy, sexual therapy, hypnotherapy, Botox, electromyography, and biofeedback (Fageeh, 2011). In a 2012 Cochrane review (Melnik *et al*., 2012), the authors reported that only 5 studies could be included, and a meta-analysis could not be performed due to group heterogeneity; and a systematic desensitization was compared with waiting control list, group therapy, in-vitro desensitization, pelvic floor exercises and hypnotherapy, but there were no clinically and no statistically significant differences.

Our aim was to investigate the effects of factors that are considered as predisposing for vaginismus on the prognosis and success rate in our clinic with cognitive-behavioral therapy and systematic desensitization exercises after sexual therapy.

## MATERIAL AND METHODS

We enrolled the patients admitted to our Sexual Function Disorders Outpatient Clinic between January 2017 and May 2018, with complaints of inability and difficulties to have sexual intercourse, and patients who were diagnosed with lifelong vaginismus and had sexual therapy with cognitive behavioral therapy. The patients’ files were scanned retrospectively, and the Ethics Committee of the Training and Research Hospital (Ethics board no. 2018/514/130/4) approved the study.

At the first visit, the patients were taken into the evaluation interviews and the individuals’ sex lives, marriage relations and family histories were questioned in detail and recorded on a standard form by the sexual therapist (also one of the investigators, A.D.A). When the male partner was not present at the first interview, he was invited to the next meeting. The patient was excluded from the study if the male partner did not come, despite the request. If it was thought that the male partner had a problem, he was referred to a urology examination, and the couple was excluded from the study only if there was no sexual intercourse due to male sexual comorbidity. After the evaluation stages, all female patients were submitted to examination in the gynecological chair in an environment where the partner was also present. Vaginismus was described as a "Genito-Pelvic Pain/ Penetration Disorder” and the patients with lifelong vaginismus were diagnosed based on “Diagnostic and Statistical Manual of Mental Disorders (DSM 5)” criteria (American Psychiatric Association, 2013). Staging was made by Lamont and Pacik system (Lamont 1-2-3-4 and Pacik 5) (Pacik & Geletta, 2017):

Lamont grade 1: Patient is able to relax for pelvic exam
Lamont grade 2: Patient is unable to relax for pelvic exam
Lamont grade 3: Buttocks lift off table. Early retreat. Toes curl upward
Lamont grade 4: Generalized retreat: Buttocks lift up, thighs close, patient retreats
Pacik grade 5: Generalized retreat as in Lamont level 4 plus visceral reaction, which may result in any one or more of the following: Palpitations, hyperventilation, sweating, severe trembling, uncontrollable shaking, screaming, hysteria, wanting to jump off the table, a feeling of going unconscious, nausea, vomiting and even a desire to attack the doctor.

After the evaluation stages, all patients diagnosed with vaginismus were treated as a couple with weekly sexual therapy, primarily with cognitive behavioral therapy technique. Meanwhile, bibliotherapy, relaxation exercises, and Kegel exercises were suggested along with sexual therapy. After explaining how to perform vaginal penetration exercises both verbally and visually on the model, the choice of site and technique was left to the couple's choice; finger (first index finger, then two fingers, first herself than her husband) or dilator (4-stage dilator, plastic) or by physician in outpatient clinic (2 fingers or 4-stage dilator). It was also suggested that the patient could start with ear stick before finger or dilator. Subsequently, vaginal penetration exercises were started for desensitization. She or her partner proposed vaginal intercourse to the couples who could manage 2-finger vaginal penetration with her partner and the penetration of 4-stage dilator. The sexual position was also left to the patient's preference; cowboy or missionary.

The collection of patient data, genital examination data, and the application of cognitive behavioral sexual therapy techniques were carried out and recorded by a single male gynecologist (A.D.A) in a private interview room. The patients were divided into three groups after therapy. Group 1 patients were those who completed vaginal penetration exercises after sexual therapy and experienced painless vaginal sexual intercourse without contraction, Group 2 involved patients who were unable to perform vaginal intercourse by failing at various stages of vaginal penetration exercises and Group 3 involved patients who were unable to start vaginal penetration exercises during sexual therapy and discontinued therapy. Demographic characteristics, vaginal penetration exercises, and factors that were thought to be vaginismus predisposing were compared among the groups.

### Statistical Analysis

We ran the statistical analysis using the SPSS 15 software program (SPSS Inc, Chicago, IL). We recorded the categorical data as number and number (%), and the continues data we recorded as mean and standard deviation. We used the Kolmogorov-Smirnov test to evaluate whether the data distribution was normal or not, and we used the parametric or nonparametric tests according to the findings. For continues data, we used the Student's t-test or Mann-Whitney U test for paired comparisons, ANOVA or Kruskal Wallis. We ran triple comparisons through a Variance Analysis, and we used the χ2 test for categorical data. *p*˂0.05 indicated statistical significance.

## RESULTS

**Figure 1:**
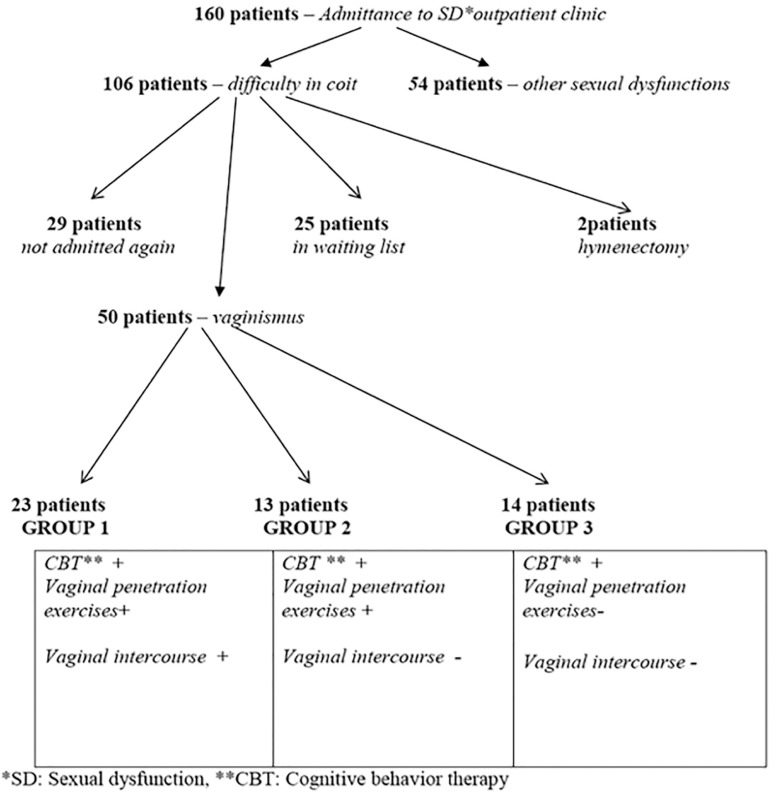
Information chart regarding patients and patient groups

There were 160 patients admitted to the Sexual Function Disorders Outpatient Clinic. Of these patients, 106 (66.2%) complained of inability to have vaginal intercourse. Of the 106 patients, 29 did not come back after the first interview; 25 entered the waiting list without starting therapy; 2 patients had hymenal septum and stenosis upon their first examination and could have vaginal intercourse after partial hymenectomy. The remaining 50 patients were considered to have vaginismus after the first interview, and they were inserted in the follow-up protocol. As a result, these 50 patients, who continued the therapy, were broken down into three groups; Group 1 (n=23), Group 2 (n=13) and Group 3 (n=14). Twenty-three (46%) patients in Group 1 completed vaginal penetration exercises following cognitive behavioral therapy, and had a painless vaginal intercourse without contraction. None of the patients had hymenal bleeding during vaginal penetration exercises. Among the successful couples, 9 (39.1%) patients were pregnant within 6 months of the treatment. Figure 1 depicts information regarding patients and groups.

The mean age of patients with vaginismus was 26.1 years, and the patients were admitted to the hospital on the 11.4 month of their marriage, in average. In our study, 22% of the patients with vaginismus were sexually abused; 64% had bad parental history; 26% had sexual disorder in the male partner; 34% had extreme addiction to mother or father and 18% had the history of vaginismus in their relatives. Seventy-six percent of patients diagnosed with vaginismus expressed that it was her fault, and 24% had psychological support or treatment. Of these patients, 78% were initially referred to the gynecologist for treatment, and 42% came to the doctor alone, but only 50% came with their partner. In their marriage, it seems that they were trying to overcome sexual problems with anal-oral sex (40%) or masturbation (78%) in general.

Table 1 depicts demographics, history and treatment parameters of the groups. According to these data, there were statistically differences between the groups in terms of history of vaginismus in relatives (4.3%, 23% and 35.7%, *p*=0.047, respectively), history of unsuccessful treatments (69%, 61% and 21.4%, *p*=0.014, respectively) and anal/oral sex ratios (47.8%, 7.7% and 57.1%, *p*=0.019, respectively).

**Table 1:** Comparison of parameters for demographics, patient’s background and treatment

*Parameters*	*Group 1 (n=23)*	*Group 2 (n=13)*	*Group 3 (n=14)*	*p*
Age (year)	26.1±3.1	25.6±5.8	26.6±3.5	*0.840^a^*
Education level (n);				
primary-secondary	2	1	1	
high school	10	8	3	*0.298^b^*
college	11	4	10	
Working condition (n) (employed/unemployed)	16/7	7/6	10/4	*0.557^b^*
Duration of marriage (month)	16.3±17.6	28±31.3	19.4±30.7	*0.420^a^*
Person she came with (n,%);				
single	11(47.8%)	5(38.5%)	5(35.7%)	
partner	10	6	9	*0.528^b^*
someone else	2	2	0	
First choice of department (n) (gynecology/others*)	13/10	13/0	13/1	***0.027**^c^*
Previous centers that were admitted (n);				
1	7	5	9	
2	10	2	3	*0.108^b^*
≥3	6	6	2	
Patients with a history of unsuccessful treatment (n,%)	16 (69%)	8 (61%)	3 (21.4%)	***0.014**^b^*
First day of admittance (month);	9.2±8.8	13.3±16.6	13.5±21.3	*0.622^a^*
>6	12	5	5	*0.557^b^*
≥12	9	5	5	*0.786^b^*
Total number of sessions	9.5±4.3	10.8±3.1	5.9±3.5	***0.003**^a^*
Duration of marriage (month);				
<12	13	6	9	
12-24	6	3	1	*0.587^b^*
>24	4	4	4	
Who was perceived as guilty (n,%);				
I am guilty	15 (65.2%)	11 (84.6%)	12 (85.7%)	*0.257^b^*
We are guilty	8	2	2	
Little or no sexual desire (n,%)	7 (30.4%)	5 (38.5%)	4 (28.6%)	*0.839^b^*
Little or no sexual pleasure (n,%)	7 (30.4%)	5 (38.5%)	4 (28.6%)	*0.839^b^*
Little or no vaginal lubrication during sexual intercourse (n,%)	4 (17.4%)	4 (30.8%)	4 (28.6%)	*0.595^b^*
Masturbation (n,%);	18 (78.3%)	9 (70.1%)	12 (85.7%)	*0.586^b^*
personal	10	4	6	*0.732^b^*
with partner	17	9	10	*0.954^b^*
Anal and/or oral sex (n,%);	11 (47.8%)	1 (7.7)	8 (57.1%)	***0.019**^b^*
anal	2	0	3	*0.172^c^*
oral	11	1	8	***0.019**^b^*
History of sexual abuse (n,%)	4 (17.4%)	3 (23.1%)	4 (28.6%)	*0.580^b^*
No experience of orgasm (n)	1	3	3	*0.191^b^*
History of bad parenting (n,%);	18 (78.3%)	6 (46.2%)	8 (57.1%)	*0.128^b^*
violence	9	3	4	*0.580^b^*
divorce	3	1	3	*0.580^b^*
infidelity	6	3	5	*0.737^b^*
repressive	9	5	4	*0.792^b^*
loveless	5	3	5	*0.618^b^*
alcoholic father	2	0	2	*0.387^c^*
Violence between partners (n);				
violence	1	1	0	*0.591^c^*
infidelity	2	0	1	*0.560^c^*
Extreme addiction to mother (n)	3	3	2	*0.717^b^*
Extreme addiction to father (n)	6	0	3	*0.136^c^*
Extreme addiction to mother of male partner (n)	3	3	2	*0,717^b^*
History of vaginismus in relatives (n,%)	1 (4.3%)	3 (23%)	5 (35.7%)	***0.047**^b^*
Psychological therapy, history of drug use (n)	4	3	5	*0.447^b^*
Stage (n,%);				*0.483^b^*
1-2	8 (34.8%)	1 (7.7%)	3 (21.4%)	
3	12	10	9	
4	3	2	2	
5	0	0	0	
Sexual disorder in male partner**	6 (26.1%)	4 (30.8%)	3 (21.4%)	*0.858^b^*

Data were presented as mean ± standard deviation (SD), number and number (%).

a Kruskal-Wallis Test (Nonparametric ANOVA),

b Chi-squared Test for Independence,

c Fisher's Exact Test,

* psychologist or psychiatrist or urologist or family doctor or spiritual person,

** premature ejaculation or late ejaculation.

There was no statistically significant difference between Groups 1 and 2 in terms of total number of sessions, but Group 3 had less number of sessions (9.5±4.3, 10.8±3.1 and 5.9±3.5, *p*=0.003, respectively) at a statistically significant level; as patients discontinued follow-up at a certain stage of therapy. Table 2 shows the comparison of treatment parameters between the exercise groups (Group 1 and Group 2). According to Table 2, there was no statistically difference between the treatment approaches and choices between the two groups.

**Table 2:** Comparison of treatment parameters between exercise groups

*Parameters*	*Group 1 (n=23)*	*Group 2 (n=13)*	*p value*
Site selection for exercise (n,%);			
at home	16 (70%)	9 (70%)	*1.000^a^*
in outpatient clinic	7 (30%)	4 (30%)	
Exercise choice (n,%);			
finger	17 (74%)	13 (100%)	
dilator	4 (17%)	0	-
penis	2 (9%)	0	
Treatment choice (n,%);			
finger in outpatient clinic	3 (13%)	4 (30%)	
finger at home	14 (61%)	9 (70%)	*0.090^b^*
other*	6 (26%)	0	
Treatment has been changed (n,%)**	8 (35%)	4 (30%)	*0.942^a^*
Patients preferred cotton swab exercise initially (n,%)	6 (26%)	4 (30%)	*0.848^a^*
Position choice in sexual intercourse attempt (n,%);			
missionary position	11 (48%)	1 (7%)	
cowboy position	12 (52%)	2 (15%)	<*0.0001^b^*
not attempted	0	10(77%)	

Data were presented as number (%).

a Fisher's Exact Test,

b Chi-squared Test for Independence,

* penis or dilator,

** transition between home/outpatient clinic and/or finger/dilator.

Table 3 shows the relationships between parameters and the number of sessions that can affect the average number of sessions in Group 1. The mean number of sessions were significantly higher in patients saying that “It is my fault” than those perceiving it as a common problem (10.6±2.9 and 7.5±5.7, *p*=0.042, respectively); and patients with sexual disorder in their male partners than those not having any problem (10 partners had premature ejaculation; 3 partners had late ejaculation) (13.3±3.7 and 8.2±3.7, *p*=0.013, respectively). There was no statistically significant difference (8.4±3.7 and 10.1±4.6, *p*=0.362, respectively) in the successful Group 1, when the treatment sessions between stage 1-2 and stage 3-4 were compared. However, although it is not meaningful, it is seen that the duration of treatment increases as the stage progresses.

**Table 3:** Parameters that might affect the mean number of sessions in successful groups and relationships between these parameters

*Parameters*		Mean number of sessions	*p* value
History of sexual abuse	Present	9.8±8.3	*0.968*
	Absent	9.5±3.3	
History of unsuccessful treatment	Present	10.6±4.2	*0.088*
	Absent	7.1±3.6	
Site selection for exercise	At home	9.7±3.4	*0.640*
	Outpatient clinic	9.1±6.2	
First day of admittance (month)	≤6	8.3±4.2	*0.355*
	>6	10.7±4.2	
Exercise preference	Finger	8.6±3.9	*0.161*
	Others*	12.2±4.6	
Starting with cotton swab	Yes	11.8±1.3	*0.079*
	No	8.7±4.7	
First choice of department	Gynecologist	8.5±3.9	*0.291*
	Other**	10.8±4.7	
Education level	≤ High school	10.2±2.6	*0.325*
	University	8.7±5.6	
Working condition	Yes	8.3±3.9	*0.061*
	No	12.3±3.9	
Extreme addiction to mother / extreme addiction to father	Yes	9.6±3.9	*0.788*
	No	9.7±4.5	
History of bad parenting***	Yes	9.3±4.5	*0.391*
	No	10.4±3.6	
Sexual desire, pleasure, vaginal lubrication	Yes	9.0±3.4	*0.592*
	No	10.3±5.5	
Anal/oral sex, masturbation	Yes	9.2±4.6	*0.356*
	No	10.6±3.3	
Whose fault is it?	My fault.	10.6±2.9	***0.042***
	Our fault.	7.5±5.7	
Position preference	Missionary position	9.3±3.6	*0.951*
	Cowboy position	9.8±4.9	
Additional treatment****	Yes	9.9±4.3	*0.518*
	No	9.3±4.5	
Psychological therapy, history of drug therapy	Present	10.8±7.4	*0.542*
	Absent	9.3±3.6	
Person she came with	Single	9.3±4.1	*0.860*
	With her partner	9.7±4.9	
Sexual disorder in male partner *****	Present	13.3±3.7	***0.013***
	Absent	8.2±3.7	
Stage	1-2	8.4±3.7	*0.401*
	≥3	10.1±4.6	

Data were presented as mean ± standard deviation (SD), number and number (%).

Statistical analysis was performed using Mann-Whitney-U test,

* dilator or penis,

** psychologist or psychiatrist or urologist or family doctor or spiritual person,

*** violence/ divorce/ infidelity/ repressive/ loveless/ alcoholism,

**** transition between at home/ outpatient clinic and/ or finger/ dilator,

***** premature ejaculation or late ejaculation.

## DISCUSSION

Although the chance of treatment success in vaginismus is theoretically 100%, this ratio is not reflected clearly in practice, because some of the patients discontinue follow-up process for a variety of reasons. The success rate was 63.8% (23/36) in patients at the exercise phase. In the literature, there are varying number of success rates, between 43% and 100%, but there are studies that show the ratio of patients discontinuing follow-up in the range of 1.2-47.8% (American Psychiatric Association, 2013; Winther *et al*., 1984; Hawton & Catalan, 1990; O'Sullivan & Barnes, 1978). As also emphasized by Schnyder *et al*. (1998), we think that continuing follow-ups increases the success rate. Why do some patients fail to reach a solution? Why do they discontinue follow-ups? Factors involving insecure therapeutic relationship with the expert, inadequate experience, choice of wrong treatment method, hidden secrets, and no male partner’s support, may affect these results. There are few studies on this subject in the literature. Yasan & Akdeniz compared successful and unsuccessful groups in their study, and they determined that the marriage age was higher in the successful group; premarital masturbation rate was also higher; the traumatic sexual experience was lower; the violence associated with vaginismus was lower; the number of patients permitting examination for determining the severity of vaginismus was lower and the number of married couples was lower without the approval of the mediators (Yasan & Akdeniz, 2009).

In our comparisons, we found that the number of previous unsuccessful treatments was higher, the preference ratio of anal/oral sex was higher, and the number of patients who were admitted to a specialist for their first visit, a gynecologist, was higher and the number of patients with vaginismus present among close relatives were lower in the successful group in comparison to other groups. Since the ratio of patients with a history of unsuccessful treatment reached 60% in Group 1 and Group 2, and it was statistically higher than Group 3, it might be a factor indicating that they were more willing to be treated in one sense. In addition, 50% of our patients were not accompanied by their husbands during their first visit. Three patients said they could not visit the clinic again because their spouses had withdrawn their support, that is, they could not find adequate spousal support.

Surveys performed on vaginismus patients in Turkey (n=2000) showed that the majority of patients were primarily seen by gynecologists or they were considering it (55%) (Turkish Sexual Health Institute). Reissing (2012) reported that gynecologists were in the lead and family physicians were the second in terms of medical visits. In our study, the ratio of first-time gynecologic visit was statistically lower in Group 1 than in Groups 2 and 3 (56.5%, 100%, 92.8%, and *p*=0.027, respectively). Could the reason be that patients in Groups 2 and 3 think that they have genital anatomic disorders rather than psychological problems because of vaginismus? According to the literature, Barnes (1986) stated that patients who though that they had anatomic disorders would complicate their treatment by ignoring their psychological bases. Likewise, Scholl (1988) noted that patients who discontinued their follow-up visits (13%) could not give up the thought of an anatomical disorder causing vaginismus and requiring surgery, which complicated the success rate of therapy. Therefore, in vaginismus patients, particularly those who visited a gynecologist for the first visit, detailed discussions should be made after an examination with vaginismus patient, whether or not there is an anatomical disorder.

In accordance with general social studies, the ratio of heterosexual oral sex among women was between 25-80%, and this ratio was between 6-32% for anal sex (Leichliter *et al*., 2007; McBride & Fortenberry, 2010). However, we could not find any scientific data regarding these ratios in patients with vaginismus, except in our study. Women with vaginismus usually cannot perform interventional procedures, such as inserting tampons or suppositories. Invasive procedures, such as needle, enema, and urethral catheter, might lead to iatrogenic vaginismus in certain patients, as the things entering them are perceived to cause violation of their bodies, and thus damage their bodies (Silverstein, 1989; Malleson, 1942). The significantly higher rates in the successful group (Group 1), in line with the general population, suggests that the ability of couples to try alternative sex routes, except vaginal intercourse, is perhaps a sense of initiative that they cannot perceive as a violation of their bodies, and contributes reaching healthy results by overcoming exercise stages in patients’ continuing treatment (Table 1). Although this condition increased the success rate of our study, it had no significant contribution to the treatment period (Table 3).

One sexual myth is the genetically transmission of vaginismus. There is no scientific evidence to prove this, but it is thought to be an acquired condition (Silverstein, 1989). Konkan *et al*. conducted their study in the Turkish population, and they found that the history of environmental and familial vaginismus (12.5%) were significantly higher in the vaginismus group than in the normal healthy population (Konkan *et al*., 2012). In our study, the presence of vaginismus in the close environment of the patients was 18%. Obviously, the presence of an individual having a history of vaginismus in a nuclear family, such as a mother or sister, or among relatives, will have a negative impact, which is already among the predisposing factors (Barnes, 1986). In our study, the number of individuals having a history of vaginismus was statistically significant among the groups, and the highest in the unsuccessful group (Group 3; 35.7%), which cannot pass to the exercise phase (Table 1). This suggests that a positive history is a predisposing factor, also negatively affecting the prognosis.

It is a general opinion that one of the important predisposing factor for vaginismus is sexual abuse. However, no significant differences were detected between the controlled groups in some controlled trials, there were studies indicating that sexual abuse was a predisposing factor; it was less than in the control group in a study (Konkan *et al*., 2012; Jeng, 2004; Basson, 1996; Brauer *et al*., 2014; Lahaie *et al.,* 2010; Reıssıng *et al.,* 2003; Dogan, 2009). In general, the history of sexual abuse was detected in vaginismus patients in the range of 2.8-28% (Konkan *et al*., 2012; Yasan & Akdeniz, 2009; Dogan, 2009; van Lankveld *et al*., 2006). In our study, we found a rate consistent with the literature, as 22% in total. In addition, we did not find any significant difference between the three groups. We also found that the presence of history of sexual abuse had no significant contribution to the treatment period (Tables 1 and 3). As in similar studies, sexual abuse does not seem to have a prognostic contribution. When we examined the statistical rates between the groups, we cannot say that there is no contribution of sexual abuse to vaginismus prognosis based only on these data.

The family structure in which the individual is raised is a significant factor for the development of several problems, including sexual dysfunctions. Repressive, frightening, threatening and extremely moral loveless parents, alcoholic father, serious arguments that can even lead to violence, and extremely protective merciful parents are common in women with vaginismus (O'Sullivan & Barnes, 1978; Silverstein, 1989; Jeng, 2004). In the study of Barnes, familial factors were present in both partners of the patient groups with vaginismus and other sexual dysfunctions (Barnes, 1986). The fact that the history of bad parenting (50%), extreme addition to mother, in which woman expressed as addictive to her mother (16%) or the conditions that were described as extreme father addition (19%), as ‘I’m my dad’s girl’, was not statistically different between the groups and did not affect the treatment period in the successful group (Tables 1 and 3). Scholl (1988) found that the treatment period was longer in the vaginismus group with sexiest negative parental behaviors. The data related to the details of familial factors are limited in the literature. Although similar familial problems are thought to be predisposing for vaginismus, there is no adequate controlled trial to prove this and to evaluate its effect on prognosis.

In our clinic, vaginal penetration exercises were explained in the method. We generally leave vaginal penetration exercises to the patient's preference. However, it is noteworthy that all the patients in Group 2 (unsuccessful exercise group) preferred exercise with their fingers. In addition, there was no significant difference in site choice among the groups (Table 2). The reason for preferring to use the finger in the unsuccessful group (100%) might be the frightening feeling of a penis-like foreign object, which means that a more tentative group may be more susceptible to failure. The literature suggests that finger exercises are as effective as dilators (Mousabi Nasab & Farnoosh, 2003), and that the dilator is generally for mild vaginismus (Saadat, 2014). Hawton & Catalan (1990) reported that they stopped treatment with the dilator because the finger had the same effect and it was more acceptable for the couple. However, bad prognostic and not choosing dilator was not mentioned in any of the studies. There are also studies showing high success rates with the dilator; in particular Masters and Johnson reported the success rate of the dilator as 98.8% (O’Donohue & Geer, 1993). In our study, the difference in site choice did not affect the successful group, but the treatment period was shorter in those who preferred the finger exercises, but it was not statistically significant (Table 3). Schnyder *et al*. (1998) compared vaginismus patients using *in vivo* (by the therapist in the outpatient clinic) and *in vitro* (at home) dilator and found that the site choice for the exercise could be left to the patient’s preference, young women who felt free would prefer exercises at home, and those who adopted the traditional approach of patient-doctor relationship may prefer to undergo the exercises in the outpatient clinic. Schnyder *et al*. (1998) recommended other alternatives, in case of an unsuccessful result, to improve success rate, as we have done in our treatment method. We also found that the ratio of treatment change was 33.3% in our study.

Factors associated with the male partners, also known as male vaginismus, are also important and are involved in the etiology (Scholl, 1988; Silverstein, 1989). The support of the male partner increased the treatment’s success rate (Barnes, 1986; Mousabi Nasab & Farnoosh, 2003; O’Donohue & Geer, 1993). Scholl (1988) noted that the treatment of couples with decisive male partners, who undertook the driving force in the treatment and cared about partner-support, was shorter. We have determined that the success rate of treatment was not changed in couples in which only women thought that it was her fault, but the treatment period was significantly prolonged in successful couples. Mutual sexual and subjective personality structures are influential in the subconscious mate selection, and vaginismus serves different purposes on both sides (Scholl, 1988; Abraham, 1956; Dawkins & Taylor, 1961; Dennerstein & Burrows, 1977). Male sexual problems are sometimes a result and sometimes a predisposing factor. In other words, vaginismus may develop in response to man's sexual problems, while vaginismus may cause sexual problems in men (Masters & Johnson, 1970; Kaplan, 1974). Dogan & Dogan (2008) detected sexual dysfunction in 65.6% of vaginismus males (50% premature ejaculation, 28.1% erectile dysfunction, 28% hypoactive sexual desire). In another study, 43.2% of man with vaginismus had sexual dysfunction (38% premature ejaculation, 8% erectile dysfunction, 5% low sexual desire). In our study, we found a total of 26% sexual dysfunctions (20% premature ejaculation, 6% late ejaculation). This ratio is almost similar to that in the general population (range; 20-30%) (Lotti & Maggi, 2018). O'Sullivan & Barnes (1978) and Scholl (1988) reported that the accompanying male’s sexual problems did not have a prognostic effect. Yasan & Akdeniz (2009) found that male sexual dysfunction did not make a difference when treated and non-treated groups were compared. We found that male sexual problems did not affect success rate, but only significantly longer treatment periods were required in successful couples (*p*<0.05).

## CONCLUSION

In conclusion, patients with vaginismus are aware of their condition and treatment is possible if they want. There is no difference between treatment methods in terms of success rates. However, the patients are more resistant to treatment if they have a history of vaginismus in their relatives or in the presence of a partner who say it is his or her fault. When we reviewed the literature, although our study had only a few number of patients, it was a preliminary work in terms of providing large data in this regard. There is a need for broader community-based prevalence studies and randomized controlled trials.
